# Ethyl 2-acetyl-3-(4-chloro­anilino)butanoate

**DOI:** 10.1107/S1600536809030876

**Published:** 2009-08-08

**Authors:** K. Rajesh, V. Vijayakumar, T. Narasimhamurthy, J. Suresh, P. L. Nilantha Lakshman

**Affiliations:** a Organic Chemistry Division, School of Science and Humanities, VIT University, Vellore 632 014, India; b Materials Research Centre, Indian Institute of Science, Bangalore 560 012, India; c Department of Physics, The Madura College, Madurai 625 011, India; d Department of Food Science and Technology, Faculty of Agriculture, University of Ruhuna, Mapalana, Kamburupitiya 81100, Sri Lanka

## Abstract

The title compound, C_14_H_18_ClNO_3_, adopts an extended conformation, with all of the main chain torsion angles associated with the ester and amino groups *trans*.  In the crystal, inversion dimers linked by pairs of N—H⋯O hydrogen bonds are observed.

## Related literature

For the crystal structure of ethyl 2-acetyl-3-anilinobutanoate, see: Priya *et al.* (2006 ▶). For hydrogen-bond motifs, see: Bernstein *et al.* (1995 ▶).
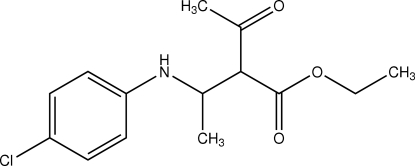


## Experimental

### 

#### Crystal data

C_14_H_18_ClNO_3_
*M* *_r_* = 283.74
Triclinic, 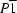

*a* = 6.9161 (2) Å
*b* = 10.1319 (3) Å
*c* = 11.4063 (3) Å
α = 87.511 (10)°
β = 80.873 (10)°
γ = 73.367 (2)°
*V* = 756.14 (4) Å^3^
*Z* = 2
Mo *K*α radiation
μ = 0.26 mm^−1^
*T* = 293 K
0.17 × 0.14 × 0.11 mm

#### Data collection

Bruker SMART APEX CCD diffractometer
Absorption correction: multi-scan (*SADABS*; Bruker, 1998 ▶) *T*_min_ = 0.958, *T*_max_ = 0.972
14994 measured reflections
4229 independent reflections
3037 reflections with *I* > 2σ(*I*)
*R*_int_ = 0.017

#### Refinement

*R*[*F* ^2^ > 2σ(*F* ^2^)] = 0.046
*wR*(*F* ^2^) = 0.132
*S* = 1.04
4229 reflections
179 parameters
H atoms treated by a mixture of independent and constrained refinement
Δρ_max_ = 0.35 e Å^−3^
Δρ_min_ = −0.31 e Å^−3^

### 

Data collection: *SMART* (Bruker, 2001 ▶); cell refinement: *SAINT* (Bruker, 2001 ▶); data reduction: *SAINT*; program(s) used to solve structure: *SHELXS97* (Sheldrick, 2008 ▶); program(s) used to refine structure: *SHELXL97* (Sheldrick, 2008 ▶); molecular graphics: *PLATON* (Spek, 2009 ▶); software used to prepare material for publication: *SHELXL97*.

## Supplementary Material

Crystal structure: contains datablocks global, I. DOI: 10.1107/S1600536809030876/ci2874sup1.cif

Structure factors: contains datablocks I. DOI: 10.1107/S1600536809030876/ci2874Isup2.hkl

Additional supplementary materials:  crystallographic information; 3D view; checkCIF report

## Figures and Tables

**Table 1:** Hydrogen-bond geometry (Å, °)

*D*—H⋯*A*	*D*—H	H⋯*A*	*D*⋯*A*	*D*—H⋯*A*
N7—H7⋯O12^i^	0.85 (2)	2.185 (19)	3.0282 (17)	170 (2)

Symmetry code: (i) 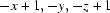.

**Fig. 1.**
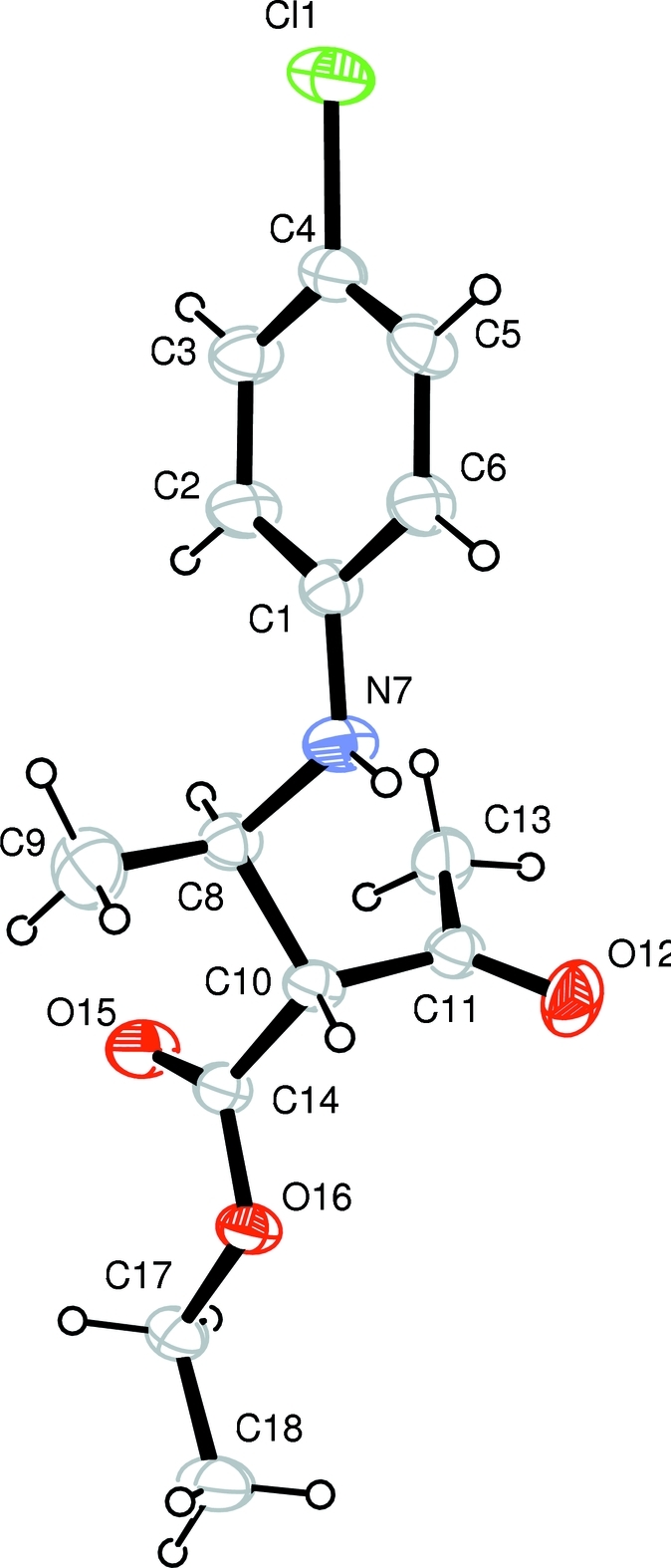
The molecular structure of the title compound, showing 30% probability displacement ellipsoids and the atom-numbering scheme.

**Fig. 2.**
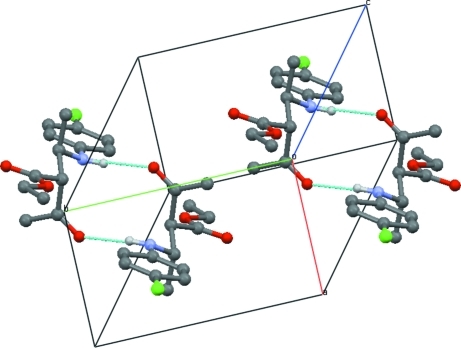
Part of the crystal structur of the title compound, showing hydrogen-bonded (dashed lines) dimers. H atoms other than H7 have been omitted for clarity.

## supplementary crystallographic information

### Comment

Ethyl butanoate is commonly used as an artificial flavoring agent in alcoholic beverages, perfumery products and as a plasticizer for cellulose. The crystal structure of ethyl 2-acetyl-3-anilinobutanoate has been reported (Priya *et al.*, 2006).

In the title molecule (Fig. 1), there are three planar subunits *viz*. the chlorophenyl amine (C1-C6/N7/Cl1), acetyl (C10/C11/O12/C13) and ethyl acetate (C10/C14/O15/O16/C17/C18) groups. The chlorophenyl amino ring is inclined at angles of 76.28 (9) and 3.48 (7)° to the acetyl and ethyl acetate groups, respectively, with the acetyl group at an angle of 72.9 (1)° to the ethyl acetate group. The molecule adopts an extended conformation, with all of the main chain torsion angles associated with the ester and amino groups, *i.e.* from C18—C17—O16—C14 to C10—C8—N7—C1 lie in the range 157.20 (14)-178.59 (15)°.

In the crystal structure, molecules associate into dimers through intermolecular N—H···O hydrogen bonds (Table 1). The hydrogen-bonded centrosymmetric dimers are characterized by an *R*_2_^2^(12) ring motif (Fig. 2) (Bernstein *et al.*, 1995).

### Experimental

A mixture of acetaldehyde (22.5 ml), ethyl acetoacetate (6.3 ml) and aniline (6.5 ml) was placed in a round bottomed flask. The contents were stirred at 273 K to 278 K for about 5 h under nitrogen atmosphere. A paste-like solid was formed, which was initially washed with benzene, then chloroform and then extracted with diethyl ether. The extract allowed to evaporate at room temperature yielded the product with crystalline nature. The resulting compound was recrystallized from diethyl ether (yield 88%, m. p. 357 K).

### Refinement

The amino H atom was located in a difference map and was refined isotropically. The remaining H atoms were placed in calculated positions and allowed to ride on their carrier atoms, with C-H = 0.93–0.98 Å and *U*_iso_(H) = 1.2*U*_eq_(C) and 1.5*U*_eq_(C_methyl_).

### Crystal data

**Table:**

C_14_H_18_ClNO_3_	*Z* = 2
*M**_r_* = 283.74	*F*(000) = 300
Triclinic, *P*1	*D*_x_ = 1.246 Mg m^−^^3^
Hall symbol: -P 1	Mo *K*α radiation, λ = 0.71069 Å
*a* = 6.9161 (2) Å	Cell parameters from 25 reflections
*b* = 10.1319 (3) Å	θ = 2–29.6°
*c* = 11.4063 (3) Å	µ = 0.26 mm^−^^1^
α = 87.511 (10)°	*T* = 293 K
β = 80.873 (10)°	Block, colourless
γ = 73.367 (2)°	0.17 × 0.14 × 0.11 mm
*V* = 756.14 (4) Å^3^	

### Data collection

**Table:**

Bruker SMART APEX CCD diffractometer	4229 independent reflections
Radiation source: fine-focus sealed tube	3037 reflections with *I* > 2σ(*I*)
graphite	*R*_int_ = 0.017
ω scans	θ_max_ = 29.6°, θ_min_ = 1.8°
Absorption correction: multi-scan (*SADABS*; Bruker, 1998)	*h* = −9→9
*T*_min_ = 0.958, *T*_max_ = 0.972	*k* = −14→13
14994 measured reflections	*l* = −15→15

### Refinement

**Table:**

Refinement on *F*^2^	Primary atom site location: structure-invariant direct methods
Least-squares matrix: full	Secondary atom site location: difference Fourier map
*R*[*F*^2^ > 2σ(*F*^2^)] = 0.046	Hydrogen site location: inferred from neighbouring sites
*wR*(*F*^2^) = 0.132	H atoms treated by a mixture of independent and constrained refinement
*S* = 1.04	*w* = 1/[σ^2^(*F*_o_^2^) + (0.0581*P*)^2^ + 0.1581*P*] where *P* = (*F*_o_^2^ + 2*F*_c_^2^)/3
4229 reflections	(Δ/σ)_max_ = 0.001
179 parameters	Δρ_max_ = 0.35 e Å^−^^3^
0 restraints	Δρ_min_ = −0.31 e Å^−^^3^

### Special details

**Table:**

Geometry. All e.s.d.'s (except the e.s.d. in the dihedral angle between two l.s. planes) are estimated using the full covariance matrix. The cell e.s.d.'s are taken into account individually in the estimation of e.s.d.'s in distances, angles and torsion angles; correlations between e.s.d.'s in cell parameters are only used when they are defined by crystal symmetry. An approximate (isotropic) treatment of cell e.s.d.'s is used for estimating e.s.d.'s involving l.s. planes.
Refinement. Refinement of *F*^2^ against ALL reflections. The weighted *R*-factor *wR* and goodness of fit *S* are based on *F*^2^, conventional *R*-factors *R* are based on *F*, with *F* set to zero for negative *F*^2^. The threshold expression of *F*^2^ > σ(*F*^2^) is used only for calculating *R*-factors(gt) *etc*. and is not relevant to the choice of reflections for refinement. *R*-factors based on *F*^2^ are statistically about twice as large as those based on *F*, and *R*- factors based on ALL data will be even larger.

### Fractional atomic coordinates and isotropic or equivalent isotropic displacement parameters (Å^2^)

**Table:**

	*x*	*y*	*z*	*U*_iso_*/*U*_eq_	
H7	0.352 (3)	0.086 (2)	0.6502 (16)	0.060 (5)*	
C1	0.3175 (2)	0.22108 (14)	0.76810 (12)	0.0460 (3)	
C2	0.2162 (3)	0.35445 (16)	0.80981 (14)	0.0591 (4)	
H2	0.1251	0.4138	0.7660	0.071*	
C3	0.2498 (3)	0.39921 (17)	0.91543 (15)	0.0602 (4)	
H3	0.1828	0.4887	0.9416	0.072*	
C4	0.3817 (3)	0.31215 (17)	0.98199 (13)	0.0524 (4)	
C5	0.4832 (3)	0.18015 (17)	0.94306 (15)	0.0572 (4)	
H5	0.5730	0.1215	0.9880	0.069*	
C6	0.4516 (2)	0.13524 (16)	0.83781 (14)	0.0540 (4)	
H6	0.5208	0.0458	0.8122	0.065*	
C8	0.1319 (2)	0.23376 (15)	0.59384 (13)	0.0484 (3)	
H8	0.0995	0.3343	0.5986	0.058*	
C9	−0.0608 (3)	0.1919 (2)	0.63979 (19)	0.0810 (6)	
H9A	−0.0328	0.0939	0.6325	0.121*	
H9B	−0.1663	0.2372	0.5941	0.121*	
H9C	−0.1051	0.2181	0.7217	0.121*	
C10	0.2170 (2)	0.18944 (13)	0.46426 (12)	0.0422 (3)	
H10	0.2416	0.0896	0.4586	0.051*	
C11	0.4189 (2)	0.22321 (14)	0.42512 (13)	0.0463 (3)	
C13	0.4247 (3)	0.36782 (16)	0.44036 (17)	0.0616 (4)	
H13A	0.4174	0.3860	0.5231	0.092*	
H13B	0.3107	0.4307	0.4105	0.092*	
H13C	0.5496	0.3796	0.3973	0.092*	
C14	0.0697 (2)	0.25923 (14)	0.37995 (12)	0.0432 (3)	
C17	−0.0456 (3)	0.23396 (18)	0.20012 (14)	0.0559 (4)	
H17A	−0.0027	0.3105	0.1618	0.067*	
H17B	−0.1881	0.2677	0.2351	0.067*	
C18	−0.0192 (3)	0.1244 (2)	0.11202 (17)	0.0780 (6)	
H18A	0.1228	0.0895	0.0799	0.117*	
H18B	−0.0958	0.1618	0.0491	0.117*	
H18C	−0.0676	0.0510	0.1500	0.117*	
Cl1	0.41769 (9)	0.37010 (6)	1.11659 (4)	0.07881 (19)	
N7	0.3003 (2)	0.17272 (14)	0.66007 (12)	0.0573 (4)	
O12	0.56910 (18)	0.13519 (11)	0.38320 (12)	0.0680 (4)	
O15	−0.03698 (19)	0.37584 (11)	0.38958 (10)	0.0631 (3)	
O16	0.07863 (16)	0.17534 (10)	0.29170 (9)	0.0495 (3)	

### Atomic displacement parameters (Å^2^)

**Table:**

	*U*^11^	*U*^22^	*U*^33^	*U*^12^	*U*^13^	*U*^23^
C1	0.0517 (8)	0.0423 (7)	0.0411 (7)	−0.0058 (6)	−0.0133 (6)	0.0036 (5)
C2	0.0749 (11)	0.0443 (8)	0.0519 (8)	0.0033 (7)	−0.0283 (8)	−0.0011 (6)
C3	0.0746 (11)	0.0474 (8)	0.0547 (9)	−0.0044 (8)	−0.0212 (8)	−0.0063 (7)
C4	0.0587 (9)	0.0610 (9)	0.0429 (7)	−0.0220 (7)	−0.0148 (6)	0.0021 (6)
C5	0.0584 (9)	0.0591 (9)	0.0549 (9)	−0.0104 (7)	−0.0259 (7)	0.0102 (7)
C6	0.0589 (9)	0.0448 (8)	0.0538 (8)	−0.0012 (7)	−0.0209 (7)	0.0029 (6)
C8	0.0551 (9)	0.0445 (7)	0.0434 (7)	−0.0071 (6)	−0.0148 (6)	0.0014 (6)
C9	0.0745 (13)	0.1022 (16)	0.0686 (12)	−0.0333 (12)	−0.0067 (10)	0.0151 (11)
C10	0.0506 (8)	0.0312 (6)	0.0456 (7)	−0.0067 (5)	−0.0186 (6)	−0.0019 (5)
C11	0.0504 (8)	0.0383 (7)	0.0478 (7)	−0.0038 (6)	−0.0150 (6)	−0.0059 (6)
C13	0.0561 (9)	0.0457 (8)	0.0833 (12)	−0.0162 (7)	−0.0042 (8)	−0.0155 (8)
C14	0.0468 (7)	0.0388 (7)	0.0446 (7)	−0.0084 (6)	−0.0151 (6)	−0.0025 (5)
C17	0.0531 (9)	0.0669 (10)	0.0467 (8)	−0.0071 (7)	−0.0219 (7)	−0.0042 (7)
C18	0.0826 (13)	0.0905 (14)	0.0631 (11)	−0.0141 (11)	−0.0310 (10)	−0.0209 (10)
Cl1	0.0954 (4)	0.0974 (4)	0.0552 (3)	−0.0350 (3)	−0.0299 (2)	−0.0060 (2)
N7	0.0747 (9)	0.0408 (7)	0.0489 (7)	0.0062 (6)	−0.0282 (6)	−0.0037 (5)
O12	0.0566 (7)	0.0485 (6)	0.0866 (9)	0.0013 (5)	−0.0009 (6)	−0.0141 (6)
O15	0.0747 (8)	0.0439 (6)	0.0632 (7)	0.0080 (5)	−0.0318 (6)	−0.0089 (5)
O16	0.0562 (6)	0.0443 (5)	0.0487 (5)	−0.0062 (4)	−0.0232 (5)	−0.0065 (4)

### Geometric parameters (Å, °)

**Table:**

C1—N7	1.3802 (18)	C10—C14	1.5173 (18)
C1—C2	1.397 (2)	C10—C11	1.526 (2)
C1—C6	1.4002 (19)	C10—H10	0.98
C2—C3	1.381 (2)	C11—O12	1.2050 (17)
C2—H2	0.93	C11—C13	1.495 (2)
C3—C4	1.374 (2)	C13—H13A	0.96
C3—H3	0.93	C13—H13B	0.96
C4—C5	1.376 (2)	C13—H13C	0.96
C4—Cl1	1.7457 (15)	C14—O15	1.1995 (16)
C5—C6	1.372 (2)	C14—O16	1.3282 (16)
C5—H5	0.93	C17—O16	1.4562 (17)
C6—H6	0.93	C17—C18	1.482 (2)
C8—N7	1.4617 (18)	C17—H17A	0.97
C8—C9	1.521 (3)	C17—H17B	0.97
C8—C10	1.537 (2)	C18—H18A	0.96
C8—H8	0.98	C18—H18B	0.96
C9—H9A	0.96	C18—H18C	0.96
C9—H9B	0.96	N7—H7	0.854 (19)
C9—H9C	0.96		
			
N7—C1—C2	123.61 (13)	C11—C10—C8	110.57 (11)
N7—C1—C6	118.80 (13)	C14—C10—H10	108.5
C2—C1—C6	117.51 (14)	C11—C10—H10	108.5
C3—C2—C1	120.73 (14)	C8—C10—H10	108.5
C3—C2—H2	119.6	O12—C11—C13	121.23 (15)
C1—C2—H2	119.6	O12—C11—C10	120.56 (13)
C4—C3—C2	120.30 (15)	C13—C11—C10	118.21 (12)
C4—C3—H3	119.9	C11—C13—H13A	109.5
C2—C3—H3	119.9	C11—C13—H13B	109.5
C3—C4—C5	120.15 (14)	H13A—C13—H13B	109.5
C3—C4—Cl1	119.42 (13)	C11—C13—H13C	109.5
C5—C4—Cl1	120.43 (12)	H13A—C13—H13C	109.5
C6—C5—C4	119.88 (14)	H13B—C13—H13C	109.5
C6—C5—H5	120.1	O15—C14—O16	124.26 (13)
C4—C5—H5	120.1	O15—C14—C10	124.71 (12)
C5—C6—C1	121.43 (14)	O16—C14—C10	111.01 (11)
C5—C6—H6	119.3	O16—C17—C18	108.05 (14)
C1—C6—H6	119.3	O16—C17—H17A	110.1
N7—C8—C9	113.62 (14)	C18—C17—H17A	110.1
N7—C8—C10	105.08 (12)	O16—C17—H17B	110.1
C9—C8—C10	112.39 (14)	C18—C17—H17B	110.1
N7—C8—H8	108.5	H17A—C17—H17B	108.4
C9—C8—H8	108.5	C17—C18—H18A	109.5
C10—C8—H8	108.5	C17—C18—H18B	109.5
C8—C9—H9A	109.5	H18A—C18—H18B	109.5
C8—C9—H9B	109.5	C17—C18—H18C	109.5
H9A—C9—H9B	109.5	H18A—C18—H18C	109.5
C8—C9—H9C	109.5	H18B—C18—H18C	109.5
H9A—C9—H9C	109.5	C1—N7—C8	124.19 (12)
H9B—C9—H9C	109.5	C1—N7—H7	114.4 (12)
C14—C10—C11	108.57 (12)	C8—N7—H7	114.5 (12)
C14—C10—C8	112.08 (11)	C14—O16—C17	116.16 (11)
			
N7—C1—C2—C3	−175.94 (17)	C8—C10—C11—O12	126.22 (15)
C6—C1—C2—C3	0.6 (3)	C14—C10—C11—C13	69.60 (16)
C1—C2—C3—C4	−0.9 (3)	C8—C10—C11—C13	−53.73 (17)
C2—C3—C4—C5	0.8 (3)	C11—C10—C14—O15	−85.49 (18)
C2—C3—C4—Cl1	−178.75 (14)	C8—C10—C14—O15	36.9 (2)
C3—C4—C5—C6	−0.3 (3)	C11—C10—C14—O16	92.66 (13)
Cl1—C4—C5—C6	179.17 (13)	C8—C10—C14—O16	−144.92 (12)
C4—C5—C6—C1	0.1 (3)	C2—C1—N7—C8	−20.5 (3)
N7—C1—C6—C5	176.54 (16)	C6—C1—N7—C8	162.99 (15)
C2—C1—C6—C5	−0.2 (3)	C9—C8—N7—C1	−79.5 (2)
N7—C8—C10—C14	−172.73 (11)	C10—C8—N7—C1	157.20 (14)
C9—C8—C10—C14	63.22 (17)	O15—C14—O16—C17	2.3 (2)
N7—C8—C10—C11	−51.45 (15)	C10—C14—O16—C17	−175.82 (12)
C9—C8—C10—C11	−175.49 (13)	C18—C17—O16—C14	−178.59 (15)
C14—C10—C11—O12	−110.45 (15)		

### Hydrogen-bond geometry (Å, °)

**Table:**

*D*—H···*A*	*D*—H	H···*A*	*D*···*A*	*D*—H···*A*
N7—H7···O12^i^	0.85 (2)	2.185 (19)	3.0282 (17)	170 (2)

Symmetry codes: (i) −*x*+1, −*y*, −*z*+1.
